# CXCR4 is a Novel Biomarker Correlated With Malignant Transformation and Immune Infiltrates in Gastric Precancerous Lesions

**DOI:** 10.3389/fmolb.2021.697993

**Published:** 2021-10-05

**Authors:** Xiaotao Jiang, Junhui Zheng, Lanxing Liu, Kailin Jiang, Yi Wen, Yanhua Yan, Yufeng Liu, Limei Zhong, Yuancheng Huang, Zhengyang Yao, Kechao Nie, Zhihua Zheng, Jinglin Pan, Peng Liu, Kunhai Zhuang, Fengbin Liu, Shijie Xu, Peiwu Li

**Affiliations:** ^1^ Department of Gastroenterology, The First Affiliated Hospital of Guangzhou University of Chinese Medicine, Guangzhou, China; ^2^ First Clinical Medical College, Guangzhou University of Chinese Medicine, Guangzhou, China; ^3^ Lingnan Medical Research Center, Guangzhou University of Chinese Medicine, Guangzhou, China; ^4^ Department of Laboratory Medicine, Guangdong Second Provincial General Hospital, Guangzhou, China; ^5^ Department of Gastroenterology, Hainan Provincial Hospital of Traditional Chinese Medicine, Haikou, China; ^6^ Baiyun Hospital of The First Affiliated Hospital of Guangzhou University of Chinese Medicine, Guangzhou, China; ^7^ Guangzhou University of Chinese Medicine, Guangzhou, China

**Keywords:** CXCR4, gastric precancerous lesions, gastric cancer, malignant transformation, immune infiltrates

## Abstract

**Background:** As early gastric cancer (EGC) has a far better prognosis than advanced gastric cancer (GC), early diagnosis and treatment are essential. However, understanding the mechanism of the process from gastric precancerous lesion (GPL) becoming EGC has made little advances. Besides, biomarkers that can monitor the progression of GPL-to-GC are still much insufficient.

**Methods:** Key gene modules associated with GPL progression to EGC were identified by integrating two GPL-related data sets, GSE55696 and GSE130823, using the WGCNA method. Combining with the TCGA-STAD cohort, hub genes were identified. Immunofluorescence was conducted to validate the expression. To explore the implication of hub genes in GPL malignant transformation, a correlation test was conducted to identify their co-expression genes, co-expression cytokines, and co-expression immune cells. Least absolute shrinkage and selection operator (LASSO) Cox regression was applied to shrink CXCR4-related predictors and construct a prognostic model. Functional enrichment was applied for exploring the potential mechanism.

**Results:** The green module in GSE55696 and the yellow module in GSE130823 were regarded as key gene modules associated with GPL progression to EGC, and 219 intersection genes from them were mainly enriched in critical immune biological processes. Combining with the TCGA-STAD cohort, CXCR4 was identified as a novel biomarker correlated with the malignant transformation of GPL, the positive rate of which was increased with GPL progression according to immunofluorescence. CXCR4 co-expression genes were found mainly involved in regulation of actin. CXCR4 co-expression cytokines were enriched in regulation of chemotaxis, cell chemotaxis, mononuclear cell migration, leukocyte chemotaxis, etc. As for co-expression immune cells, the expression level of CXCR4 was positively correlated with the abundance of macrophages but negatively correlated with that of effector memory T cells and NKT cells during GPL malignant transformation. In addition, the CXCR4-related prognostic model was able to predict the prognosis of GC and serve as an independent predictor for overall survival (OS).

**Conclusions:** CXCR4 was a novel biomarker correlated with malignant transformation of GPL and played a vital role in the control of tumor immunity. CXCR4 is possible to serve as a therapeutic target for malignant transformation of GPL.

## Introduction

Gastric cancer (GC) is the fifth most common malignancy and the third leading cause of cancer-related mortality (Freddie et al., 2018). Histologically, Lauren categorized GC into two subtypes: intestinal and diffuse types (Laurén, 1965). Intestinal-type GC (IGC), with a clear and multistep histological evolution starting from chronic inflammation and progressing to atrophy, intestinal metaplasia, gastric precancerous lesion [GPL, including low-grade intraepithelial neoplasia (LGIN) and high-grade intraepithelial neoplasia (HGIN)], and frank malignancy (Correa, 1988), and diffuse-type GC (DGC), without having to go through the Correa pathway, directly become cancerous through highly active inflammation (Nardone et al., 2004; Watanabe et al., 2012). As early gastric cancer (EGC) has a far better prognosis than advanced GC, early diagnosis and treatment are essential (Gotoda et al., 2000). However, biomarkers that can monitor the progression of GPL-to-GC are still much insufficient. The relatively fixed evolutionary paradigm of IGC makes it possible to develop biomarkers to monitor the early onsets.

With the development of public databases such as the Gene Expression Omnibus (GEO) database, huge amounts of genomic, epigenomic, transcriptional, and proteomic data become easier to access (Barrett et al., 2013). Correspondingly, various algorithms designed for analyzing omics data have also been developed over the past few years (Lin and Lane, 2017), which provide more opportunities to explore the molecular mechanism and biomarkers of carcinogenesis.

Recently, cancer immunotherapy has been a research “hot spot”. CXC chemical receptors (CXCRs), as a key component of the immune system, have received increasing attention. Belonging to a subfamily of G-protein coupled receptors, CXCR family members (comprising CXCR1-7) serve as crucial regulators of cancer progression through binding to the corresponding ligands (Ben-Baruch, 2006; Lee et al., 2014). CXCRs and their ligands could facilitate tumor cell activation, proliferation, invasion, and migration (Zhu et al., 2012). In addition, CXCR-associated angiogenensis and tumor infiltrating immune cells also attracted more and more attention.

Herein, we carried out a series of bioinformatics analysis on GPL and GC-related data sets and identified CXCR4 as a novel biomarker correlated with malignant transformation, which was also validated by immunofluorescence. We also explored the immune implication of CXCR4 in GPL malignant transformation and constructed a CXCR4-related prognostic model.

## Methods

### Data Acquisition and Preprocessing

Raw data of two GPL-related gene expression profiles [GSE55696 (Xu et al., 2014) and GSE130823 (Zhang et al., 2020)] were downloaded from the GEO database on NCBI (www.ncbi.nlm.nih.gov/geo/). Then, they were processed and normalized using tools from the limma package (Smyth et al., 2010) (the R code was provided in the supplementary file). The annotaion function in Sangerbox (http://soft.sangerbox.com/) software was used to annotate the probe. GSE55696 performed on an Agilent-014850 Whole Human Genome Microarray 4 × 44K G4112F includes 19 LGIN, 20 HGIN, 19 EGC, and 19 chronic gastritis (CG) samples. GSE130823 performed on an Agilent-039494 SurePrint G3 Human GE v2 8 × 60K Microarray 039381 includes 17 LGIN, 14 HGIN, 15 EGC, and 47 paired inflammation controls. In order to investigate the difference between individuals from different pathological stages, data of 47 paired inflammation controls in GSE130823 were excluded. As stomach adenocarcinoma (STAD) is the most common form of GC and regarded as the final stage of the Correa cascade (Bockerstett and Dipaolo, 2017), RNA sequencing data of STAD samples with clinical information in TCGA were adopted and downloaded from the UCSC Xena browser (https://xenabrowser.net/) (Goldman et al., 2015). After removing samples with 0-day follow-up duration and incomplete clinical information, 350 STAD samples and 32 adjacent samples were obtained. Among 350 STAD samples, 46 were in Stage I, 110 were in Stage II, 145 were in Stage III, 35 were in Stage IV, and 14 were unknown.

### Identification of Key Gene Modules Associated With GPL Progression to EGC

To identify key gene modules associated with GPL progression, weighted gene co-expression analysis (WGCNA) was used to construct a co-expressed gene network and explore its correlation with clinical traits. First, through utilizing the “WGCNA” package in R (Langfelder and Horvath, 2008), top 25% genes with the largest variance differences were used to construct weight gene co-expression networks in GSE55696 and GSE130823 data sets. Then, a similarity matrix was established based on the Pearson’s correlation value between the paired genes. Next, an adjacency matrix was created using the formula (Zhang and Horvath, 2005) a_mn_ = |c_mn_|^β^ (a_mn_: adjacency matrix between gene m and gene n, c_mn_ = Pearson’s correlation between paired genes, β: soft power value) and converted into a topological overlap matrix. The value of soft threshold power was chosen with the scale-free topology scale R^2^ exceeding 0.85. Genes with similar expression patterns were categorized into modules by average linkage hierarchical clustering with the module minimum size set as 100, and colors were used to label modules. Those genes that cannot be assigned to any of the modules were placed in a gray module. That is, the genes in the gray module were not co-expressed. To explore the correlation between gene modules and clinical traits, we defined “Stage” representing the severity of pathology, in which CG was represented by “1,” LGIN by “2,” HGIN by “3,” and EGC by “4.” Then, the correlation between module eigengenes (MEs) and the clinical traits including “Stage” were calculated using Pearson’s correlation test in GSE55696 and GSE130823 data sets, with a *p*-value < 0.05 as the cutoff. Those gene modules harboring the highest correlation coefficient with “Stage” were considered as key gene modules associated with GPL progression to EGC.

### Identification of Novel Genes Associated With GPL Progression

Among the intersecting genes of key gene modules from GSE55696 and GSE130823 data sets, we regarded that novel genes should be differentially expressed between the tumor and adjacent normal tissue and significantly correlated with prognosis. Therefore, novel genes were identified with the following three criteria met meanwhile in the TCGA-STAD cohort: 1) significantly associated with survival (filtered by Kaplan–Meier (K-M) survival curves utilizing the “survival” package with a Log-rank *p*-value < 0.05 as the cutoff), 2) harboring a predictive ability for overall survival (OS) with the area under the curve (AUC) ≥0.60 (utilizing the “timeROC” package), 3) differently expressed between the tumor and adjacent normal tissue (utilizing the linear model and the empirical Bayes method in the “limma” package with |logFC|≥0.585 and a false discovery rate (FDR) < 0.05 as the cutoff).

### Patients and Stomach Tissues

A total of 13 stomach tissue samples including 4 CG, 4 LGIN, 3 HGIN, and 3 GC were obtained from the patients treated at the First Affiliated Hospital of Guangzhou University of Chinese Medicine. All these samples were stored at −80°C. All patients signed informed consents before operation. The study was approved by the Ethics Committee of the First Affiliated Hospital of Guangzhou University of Chinese Medicine.

### Validation of CXCR4 Expression Based on Immunofluorescence

Stomach tissues were fixed in 4% paraformaldehyde, embedded in paraffin, and sectioned into five mum slices for the immunofluorescence assay. The slice was then put into dimethylbenzene xylene I for 15 min, dimethylbenzene xylene II for 15 min, ethanol I for 5 min, anhydrous ethanol II for 5 min, 75% alcohol for 5 min, 85% alcohol for 5 min, and distilled water in turn. Then, the slice was put into a repair box which was filled with the ethylenediaminetetraacetic acid antigen repair buffer (pH 8.0). The antigen repair was performed in a microwave with the medium boiling for 5 min. After natural cooling, the slice was placed into phosphate-buffered saline (PBS) (pH 7.4) and washed on a decoloring table three times, 5 min each time. After spin-drying, a circle was drawn on the slice with a Pap Pen to prevent the antibody from running away. Dropping 3% bovine serum albumin in the circle, the tissue was covered and closed for 30 min at room temperature. The sealing solution was gently shaken off, PBS was dropped on the slice to prepare the first antibody in a certain proportion, and the slice was laid flat in the wet box at 4°C for incubation overnight. Then, the slice was put into PBS (pH 7.4) for decoloration (washing three times, 5 min each time). After slightly drying, the secondary antibody with the corresponding species of the primary antibody was added dropwise in the circle to cover the tissue and incubated for 50 min at room temperature in the dark. The slide was put in PBS (pH7.4), and it was shaken on the bleaching shaker three times, each time for 5 min. After spin-drying, the slice was added with 4,6-diamidino-2-phenylindole and incubated for 10 min at room temperature in the dark. After being washed three times and sealed, the slice was inverted on a fluorescence microscope to collect images. The stained sections were scanned with a panoramic scanner (panoramic MIDI II, 3DHISTH). Quantification of the immunostained images generated was carried out using HistoQuant software (Quant Center 2.0 software, 3DHISTH). The percentage of the positively stained area was obtained by dividing the stained area by the total tissue area, multiplied by 100.

### Identification of CXCR4 Co-expression Genes

The Spearman correlation test was conducted to identify genes significantly correlated with CXCR4 expression in GPL-related cohorts (GSE55696 and GSE130823) and the GC cohort (TCGA-STAD), with a *p*-value < 0.05 as the cutoff. Intersecting CXCR4-related genes from the above three cohorts were considered as crucial CXCR4 co-expression genes.

### Identification of CXCR4 Co-expression Cytokines

Annotation 456 cytokines were downloaded from ImmPort Portal (https://www.immport.org/). Gene expression data of cytokines in GSE55696, GSE130823, and TCGA-STAD cohorts were obtained, and the correlations with the CXCR4 expression level were calculated based on the Spearman correlation test with a *p*-value < 0.05 as the cutoff. Similarly, intersecting co-expressed cytokines from the above three cohorts were regarded as crucial CXCR4 co-expression cytokines.

### Identification of CXCR4 Co-expression Immune Cells

The Immune Cell Abundance Identifier (ImmuCellAI, http://bioinfo.life.hust.edu.cn/web/ImmuCellAI/) is a gene set signature-based method for precisely estimating the abundance of 24 immune cell types, especially T-cell properties (18 T-cell subsets) (Miao et al., 2020). It is reported that it has the best performance in immune cell abundance estimation (Miao et al., 2020), compared with other five methods [xCell (Aran et al., 2017), CIBERSORT (Newman et al., 2015), EPIC (Racle et al., 2017), MCP-counter (Becht et al., 2016), and TIMER (Li et al., 2017)]. Gene expression data of GSE55696, GSE130823, and TCGA-STAD cohorts were submitted to ImmuCellAI to acquire the estimation of immune cell abundance. Likewise, the Spearman correlation test was also conducted to identify immune cells co-expressed with CXCR4 based on a *p*-value < 0.05 as the cutoff. Intersecting co-expressed immune cells of CXCR4 from the above three cohorts were regarded as crucial CXCR4 co-expression immune cells.

### Construction of the Protein–Protein Interaction (PPI) Network

Target genes were uploaded to STRING (http://string-db.org) to acquire the information about interactions, with a limitation of the species to “*Homo sapiens*” and a confidence score greater than 0.4.

### Functional Enrichment Analysis

The enrichment analysis of Gene Ontology (GO) was carried out according to target genes to explore potential molecular mechanisms by utilizing the “clusterProfiler” R package. The *p*-values are adjusted using the BH method to control the FDR.

### Construction of a CXCR4-Related Prognostic Model

In the TCGA-STAD cohort, the LASSO Cox regression analysis by the R package “glmnet” was performed to select the valuable prognostic markers from the co-expression genes of CXCR4 and the optimal penalty parameter (λ) was estimated by tenfold cross-validation to prevent overfitting. The risk score of each sample was determined by the following formula: risk score ∝ e^sum(each gene’s expression × corresponding coefficient)^. The patients were separated into high- and low-risk groups based on the optimal cutoff values. A K-M survival curve and time-dependent ROC analysis were conducted to compare the survival and evaluate the model’s predictive ability, respectively. Survival analysis was implemented within the “survivalROC” R package. Univariate and multivariate Cox regression analyses were used to determine the independent predictors for OS.

### Cell Culture

Purchased from the American Type Culture Collection (ATCC, Manassas, VA, USA), GC cell line AGS and normal human gastric epithelial cell line GES-1 were cultured in the RPMI-1640 Medium (Life Technologies, Grand Island, NY, USA) supplemented with 10% fetal bovine serum (Life Technologies) at 37°C in a humidified atmosphere with 5% CO_2_.

### Quantitative Real-Time Polymerase Chain Reaction

Total RNA was extracted from cells with the TRIzol reagent (Invitrogen, China) according to the manufacturer’s protocol. Reverse transcription was performed according to the manufacturer’s instructions using a PrimeScript RT Reagent Kit (Takara, China). A SYBR PrimeScript RT-PCR Kit (Takara) was applied for the analysis of quantitative reverse transcription-polymerase chain reaction (qRT-PCR). The 2^−ΔΔCt^ statistic was used to calculate the expression levels of genes.

### Statistical Analysis

Student’s t test or the Mann–Whitney test was used to compare gene expression levels of different groups depending on the distribution of data. Pearson’s or Spearman’s correlation tests were performed in order to evaluate statistical correlation depending on the distribution of data. One-way analysis of variance (ANOVA) was used to perform a comparison for continuous variables among groups ≥3. The OS between groups was compared by using the Kaplan–Meier analysis with the log-rank test. Also, the identification of independent predictors of OS was conducted by the analysis of univariate and multivariate Cox regression. All statistical analyses were performed with R software (Version 3.6.3) or GraphPad Prism software (Version 8.0). All *p*-values are two-tailed, and a *p*-value less than 0.05 was considered statistically significant.

## Results

### Key Gene Modules Associated With GPL Progression to EGC

**FIGURE 1:**
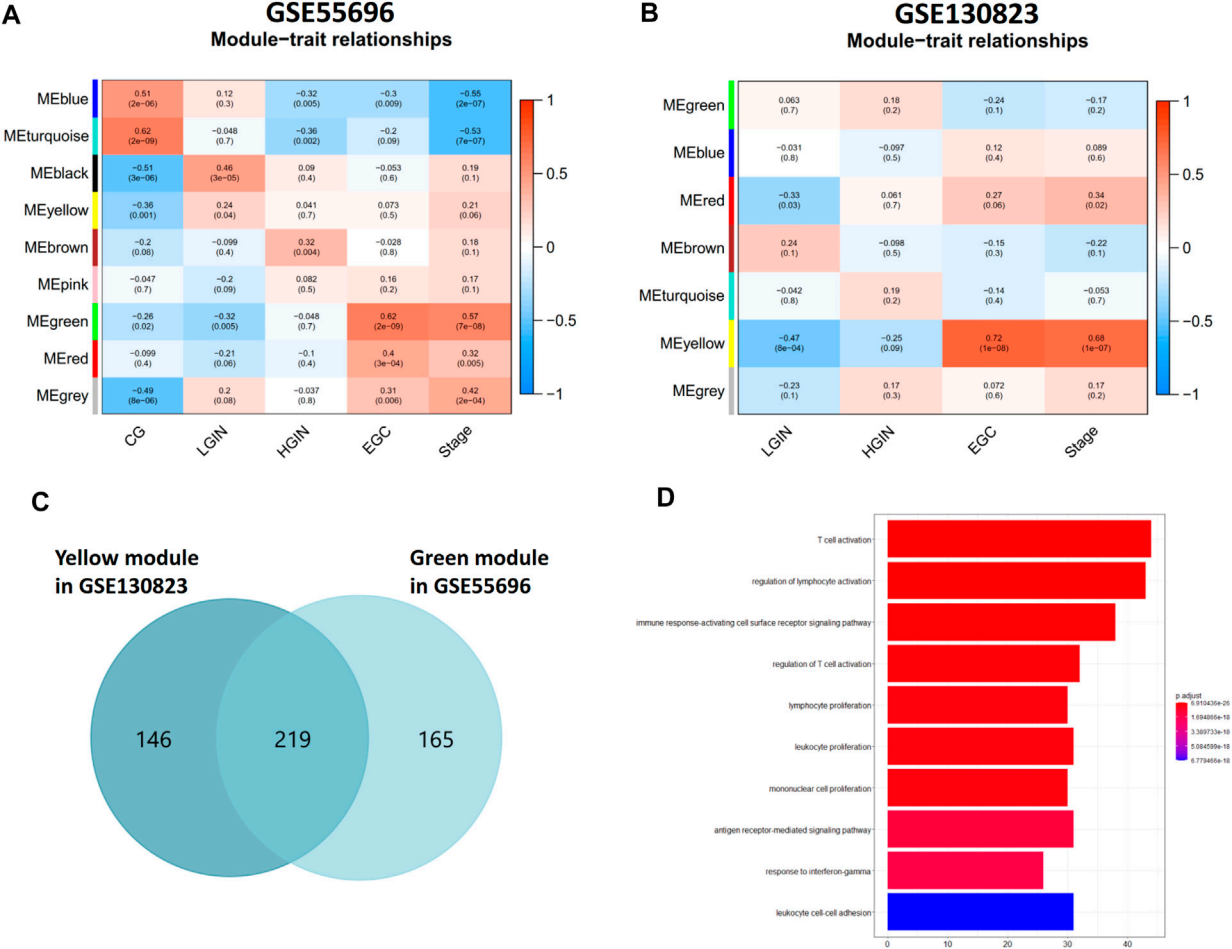
Identification of key gene modules associated with GPL progression to EGC. **(A, B)** Module-trait relationships in the GSE55696 cohort and GSE130823 cohort. Each row corresponds to a color module, and each column correlates to the clinical trait. **(C)** Venn diagram showing 219 intersection genes from the green module in GSE55696 and the yellow module in GSE130823, which were regarded as key gene modules associated with GPL progression to EGC. **(D)** Results of GO enrichment of the 219 intersection genes.

A total of 4,359 genes in GSE55696 and 8,020 genes in GSE130823 with the highest expression variance (top 25%) were selected for subsequent WGCNA. β = 6 (scale-free R^2^ = 0.87) and β = 16 (scale-free R^2^ = 0.85) were the lowest power fit scale-free indices over 0.85 and determined as the soft-thresholding power parameters to ensure a scale-free network in GSE55696 and GSE130823, respectively (Supplementary Figure S1). Eventually, genes with similar expression patterns were grouped into 9 and 7 co-expression modules, respectively, in GSE55696 and GSE130823 (Figures 1A,B). The gray module is a set of genes that cannot be clustered to any module. We found that the green module (R = 0.57, p = 3e-07) in GSE55696 and the yellow module (R = 0.68, p = 1e-07) in GSE130823 showed the highest correlation with Stage. Regarding as key gene modules associated with GPL progression to EGC, the green module in GSE55696 possessed 365 genes, while the yellow module in GSE130823 harbored 384 genes. Venn diagrams obtained 219 intersection genes from the above two key modules (Figure 1C), and they were mainly enriched in critical immune biological processes, including T-cell activation, regulation of lymphocyte activation, the immune response-regulating cell surface receptor signaling pathway, and so on (Figure 1D).

### Identification and Validation of Novel Genes Associated With GPL Progression to EGC

**FIGURE 2:**
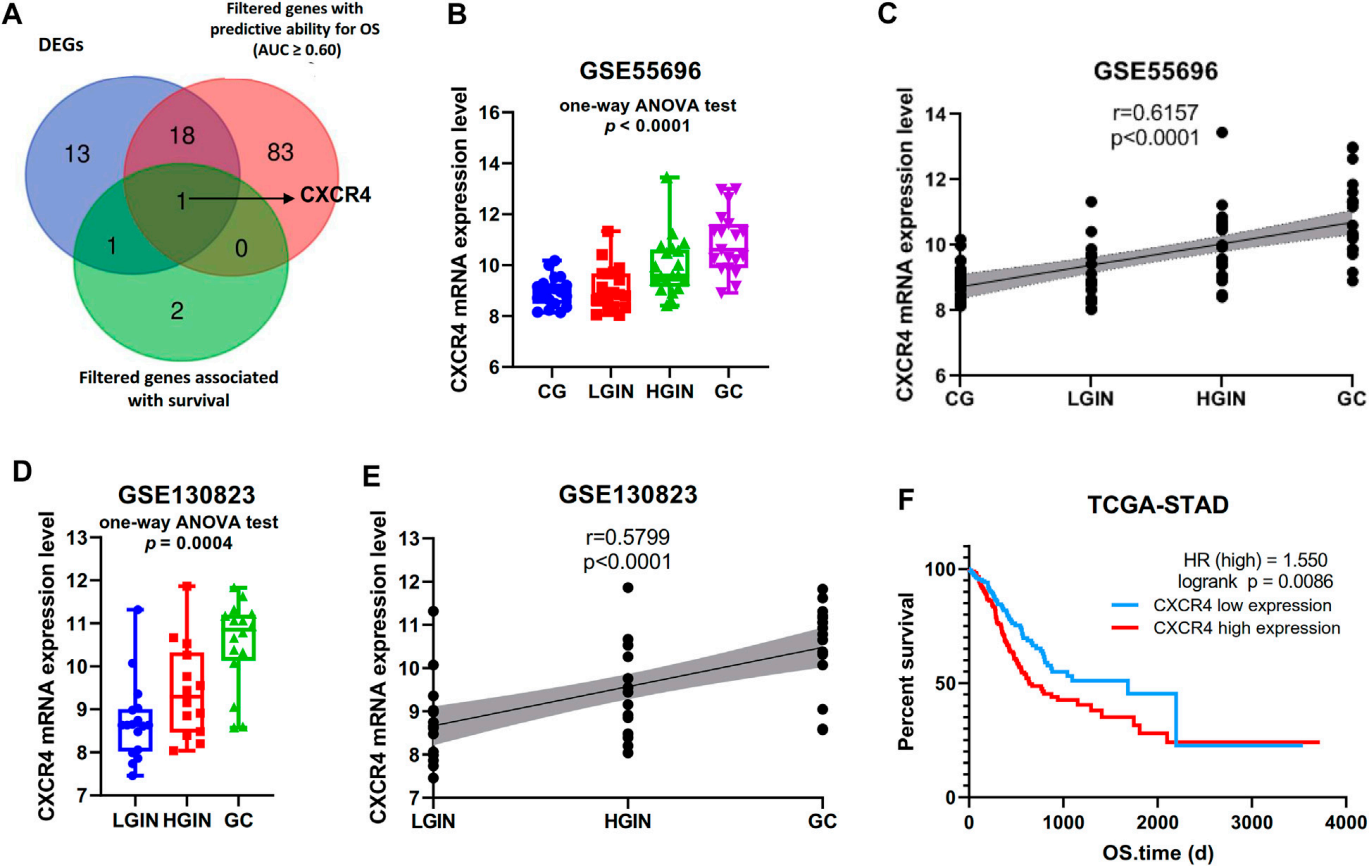
Identification of novel genes associated with GPL progression to EGC. **(A)** Venn diagram showing that CXCR4 was the novel gene. **(B, D)** Gene expression level of CXCR4 in the pathological stage in GSE55696 and GSE130823 cohorts. **(C, E)** Scatter plot showing the correlation between CXCR4 expression levels and the pathological stage in GSE55696 and GSE130823 cohorts. **(F)** Survival analysis of CXCR4 in the TCGA-STAD cohort. The patients were stratified into the high-level group and low-level group according to the median expression level.

**FIGURE 3:**
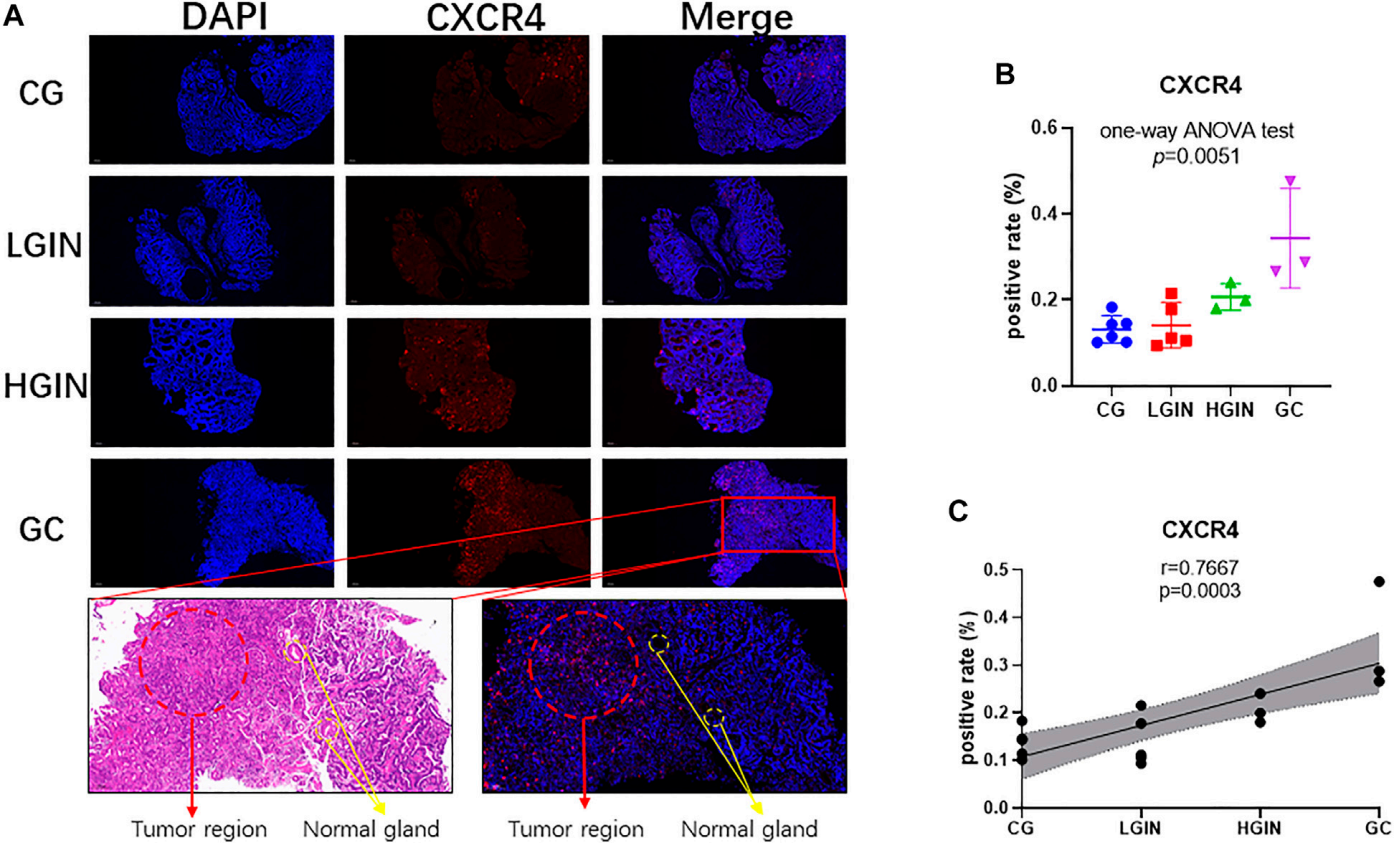
Expression of CXCR4 in different pathological stages of stomach tissues. **(A)** Immunofluorescence (scale bar = 200 μm). **(B)** Gene expression level of CXCR4 in the pathological stage. **(C)** Scatter plot showing the correlation between CXCR4 expression levels and the pathological stage.

Among the 219 intersection genes, 33 genes were differentially expressed between the tumor and adjacent normal tissue, 102 genes harbored predictive ability for OS with AUC ≥0.60, and 4 genes were significantly associated with survival. After an intersection, CXCR4 were found to meet the above three criteria meanwhile (Figure 2A). Expression profile data showed that CXCR4 expression was a significant difference among pathological stages (GSE55696: *p* in the one-way ANOVA test <0.0001, Figure 2B; GSE130823, *p* in the one-way ANOVA test = 0.0004, Figure 2D) and significantly positively correlated with tumorigenesis (GSE55696: r = 0.6157, *p* < 0.0001, Figure 2C; GSE130823, r = 0.5799, *p* = 0.0001, Figure 2E). Survival analysis indicated that the increased expression of CXCR4 was significantly associated with poorer prognosis (Figure 2F, *p* = 0.0086). We validated CXCR4 expression based on immunofluorescence in 13 stomach tissue samples including 4 CG, 4 LGIN, 3 HGIN, and 3 GC. The positive rate of CXCR4 was also a significant difference among pathological stages (*p* in the one-way ANOVA test = 0.0051, Figures 3A,B) and significantly positively correlated with tumorigenesis (r = 0.7667, *p* = 0.0003, Figure 3C). We also observed that CXCR4 mainly expressed inside the tumor region but rarely in the peri-tumor (Figure 3A).

### CXCR4 Co-expression Genes

**FIGURE 4:**
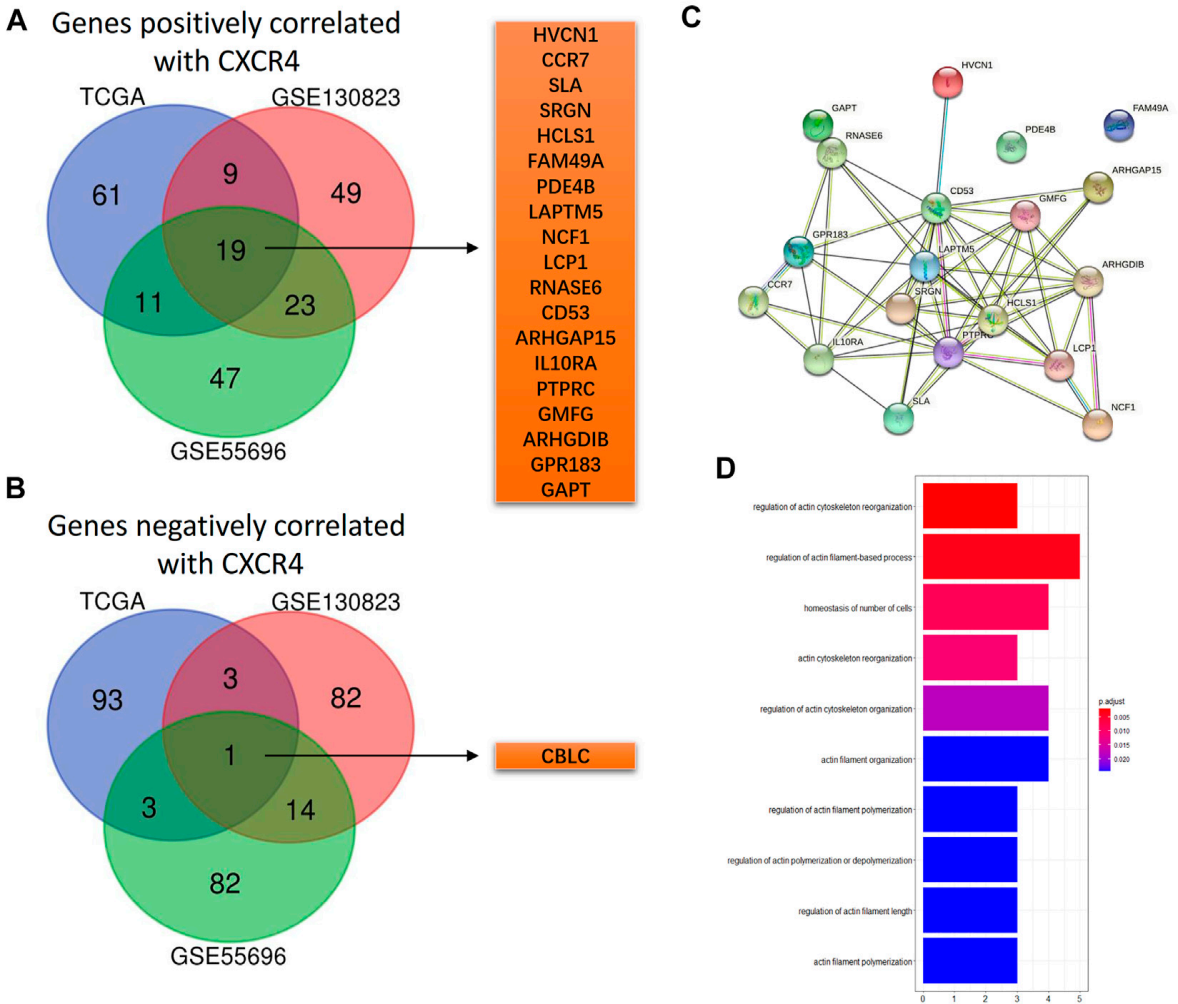
CXCR4 co-expression genes. **(A)** Venn diagram showing 19 shared genes positively correlated with CXCR4 from GSE55696, GSE130823, and TCGA cohorts. **(B)** Venn diagram showing one shared gene negatively correlated with CXCR4 from GSE55696, GSE130823, and TCGA cohorts. **(C)** PPI network of the 19 genes positively correlated with CXCR4. **(D)** Results of GO enrichment of the 19 genes positively correlated with CXCR4.

According to the Spearman correlation test, there were 19 genes positively correlated with CXCR4 (HVCN1, CCR7, SLA, SRGN, HCLS1, FAM49A, PDE4B, LAPTM5, NCF1, LCP1, RNASE6, CD53, ARHGAP15, IL10RA, PTPRC, GMFG, ARHGDIB, GPR183, and GAOT, Figure 4A), and the interaction network is shown in Figure 4C. However, only one gene was negatively correlated with CXCR4 (Figure 4B). According to functional enrichment, the 19 genes positively correlated with CXCR4 were mainly involved in regulation of actin (Figure 4D).

### CXCR4 Co-expression Cytokines

**FIGURE 5:**
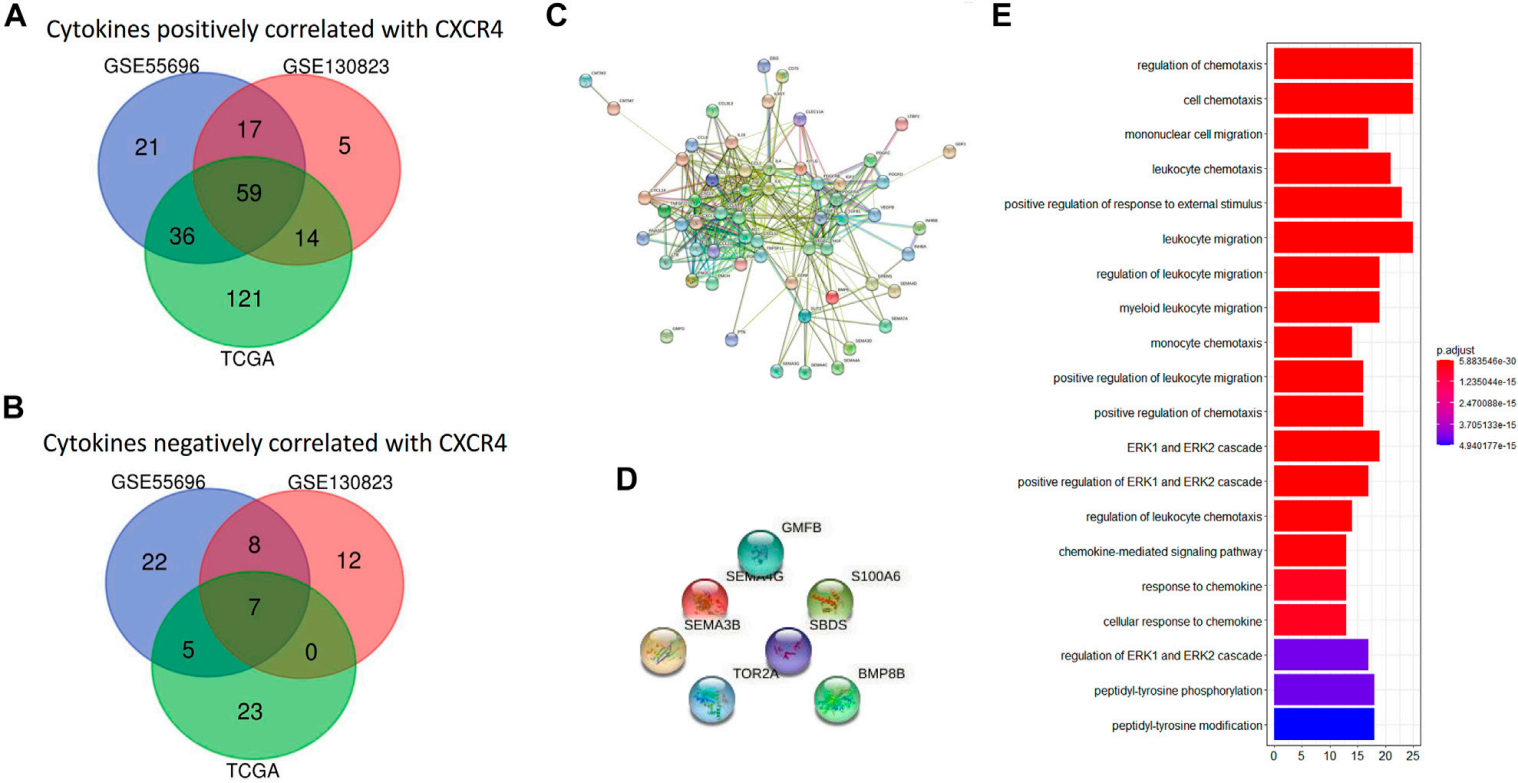
CXCR4 co-expression cytokines. **(A)** Venn diagram showing 59 shared cytokines positively correlated with CXCR4 from GSE55696, GSE130823, and TCGA cohorts. **(B)** Venn diagram showing seven shared cytokines negatively correlated with CXCR4 from GSE55696, GSE130823, and TCGA cohorts. **(C)** PPI network of the 19 genes positively correlated with CXCR4. **(D)** PPI network showed no interaction between seven cytokines negatively correlated with CXCR4. **(E)** Results of GO enrichment of the 59 cytokines positively correlated with CXCR4.

In order to explore the immune implication of CXCR4, CXCR4 co-expression cytokines were identified. The number of cytokines positively correlated with CXCR4 was up to 59, and their interaction was significant (Figures 5A,C). However, there are only seven cytokines negatively correlated with CXCR4, and no significant interaction was observed (Figures 5B,D). Figure 5E shows the predominate biological processes of CXCR4 positively related cytokines such as regulation of chemotaxis, cell chemotaxis, mononuclear cell migration, leukocyte chemotaxis, etc.

### CXCR4 Co-expression Immune Cells

**FIGURE 6:**
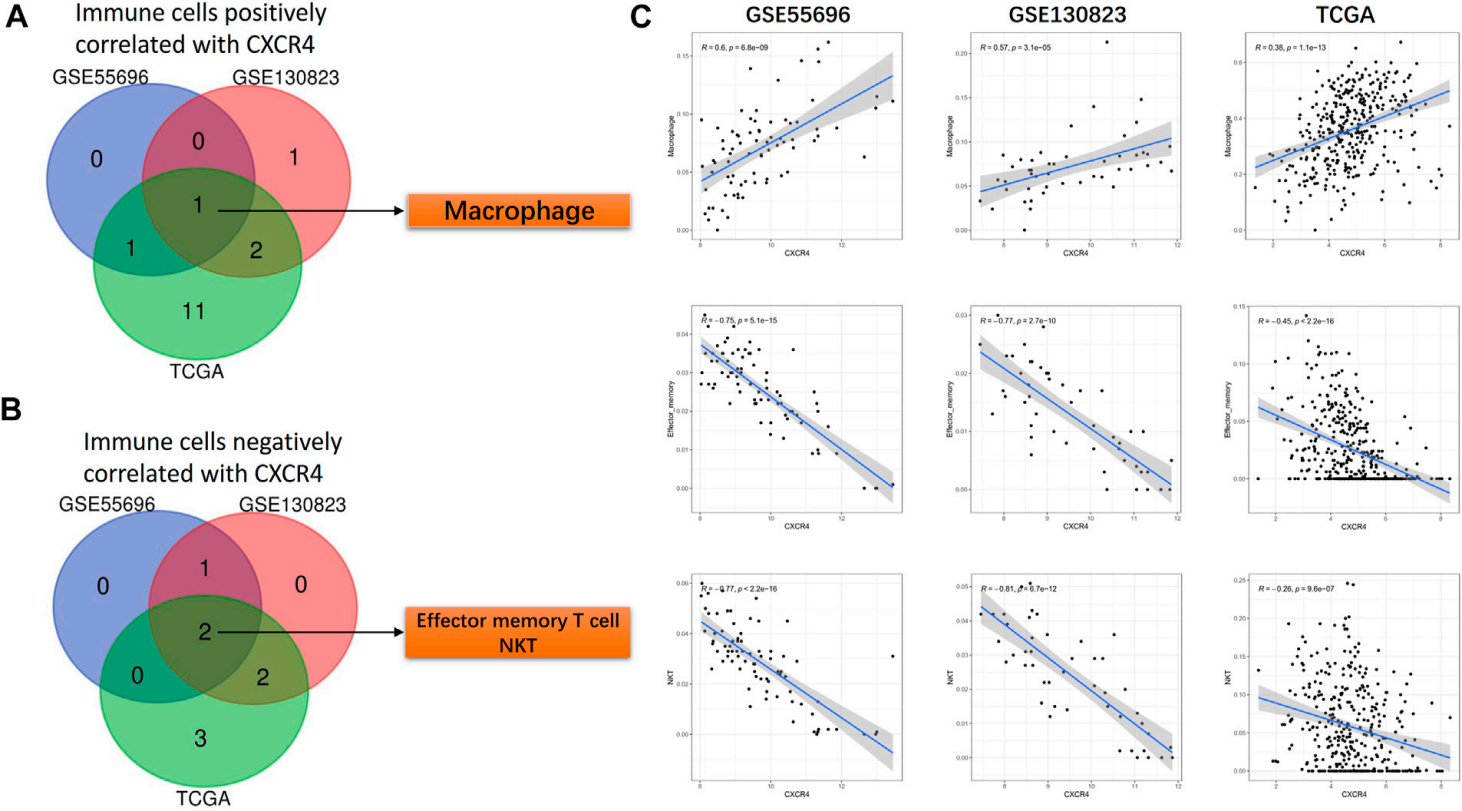
CXCR4 co-expression immune cells. **(A)** Venn diagram showing macrophages positively correlated with CXCR4 expression in GSE55696, GSE130823, and TCGA cohorts. **(B)** Venn diagram showing effector memory T cells and NKT cells negatively correlated with CXCR4 expression in GSE55696, GSE130823, and TCGA cohorts. **(C)** Scatter plot showing the correlation between macrophages, effector memory T cells, and NKT cell abundance and CXCR4 expression levels in GSE55696, GSE130823, and TCGA cohorts.

In a similar manner, we identified CXCR4 co-expression immune cells. In GPL and GC cohorts, the expression level of CXCR4 was positively correlated with the abundance of macrophages (GSE55696: r = 0.6, *p* = 6.8e-09, GSE130823: r = 0.57, *p* = 3.1e-05, TCGA: r = 0.38, *p* = 1.1e-13, Figure 6A,C) but negatively correlated with that of effector memory T cells (GSE55696: r = -0.75, *p* = 5.1e-15, GSE130823: r = −0.77, *p* = 2.7e-10, TCGA: r = −0.45, *p* < 2.2e-16, Figures 6B,C) and NKT cells (GSE55696: r = −0.77, *p* < 2.2e-16, GSE130823: r = −0.81, *p* = 6.7e-12, TCGA: r = −0.26, *p* = 9.6e-07, Figures 6C).

### Construction of a CXCR4-Related Prognostic Model

**FIGURE 7:**
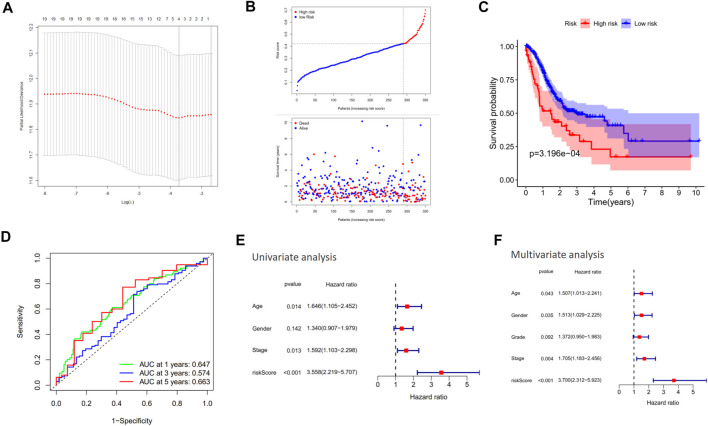
Construction of a CXCR4-related prognostic model. **(A)** Cross-validation for tuning parameter selection in the proportional hazard model. **(B)** Distribution of risk score in the TCGA cohort. **(C)** Kaplan–Meier survival analysis of OS between the high-risk group and low-risk group in the TCGA cohort. **(D)** AUC in ROC analysis for risk signature at 1-, 3-, and 5-year survival time in the TCGA cohort. **(E, F)** Results of the univariate and multivariate Cox regression analyses of OS in the TCGA cohort.

Among the 20 co-expression genes of CXCR4, the LASSO Cox regression analysis identified 4 predictors (i.e., FAM49A, GPR183, CCR7, and GAPT) with the greatest impact on the OS of GC patients (Figure 7A). Based on the optimal cutoff of risk score, the patients were divided into high-risk (*n* = 60) and low-risk (*n* = 290) groups and the patients with a higher risk score tended to possess a poorer survival (Figure 7B). The K-M curve revealed that patients in the high-risk group had worse survival outcomes compared to those in the low-risk group (Figure 7C, *p* = 3.196e-04) and the AUC of ROC for 1-, 3-, and 5-year OS reached 0.647, 0.574, and 0.663, respectively (Figure 7D). Univariate and multivariate analyses also confirmed the independent prognostic value of this model (Figures 7E,F).

### Validation of Novel Gene Expression Levels and Correlationship in Cell Lines

**FIGURE 8:**
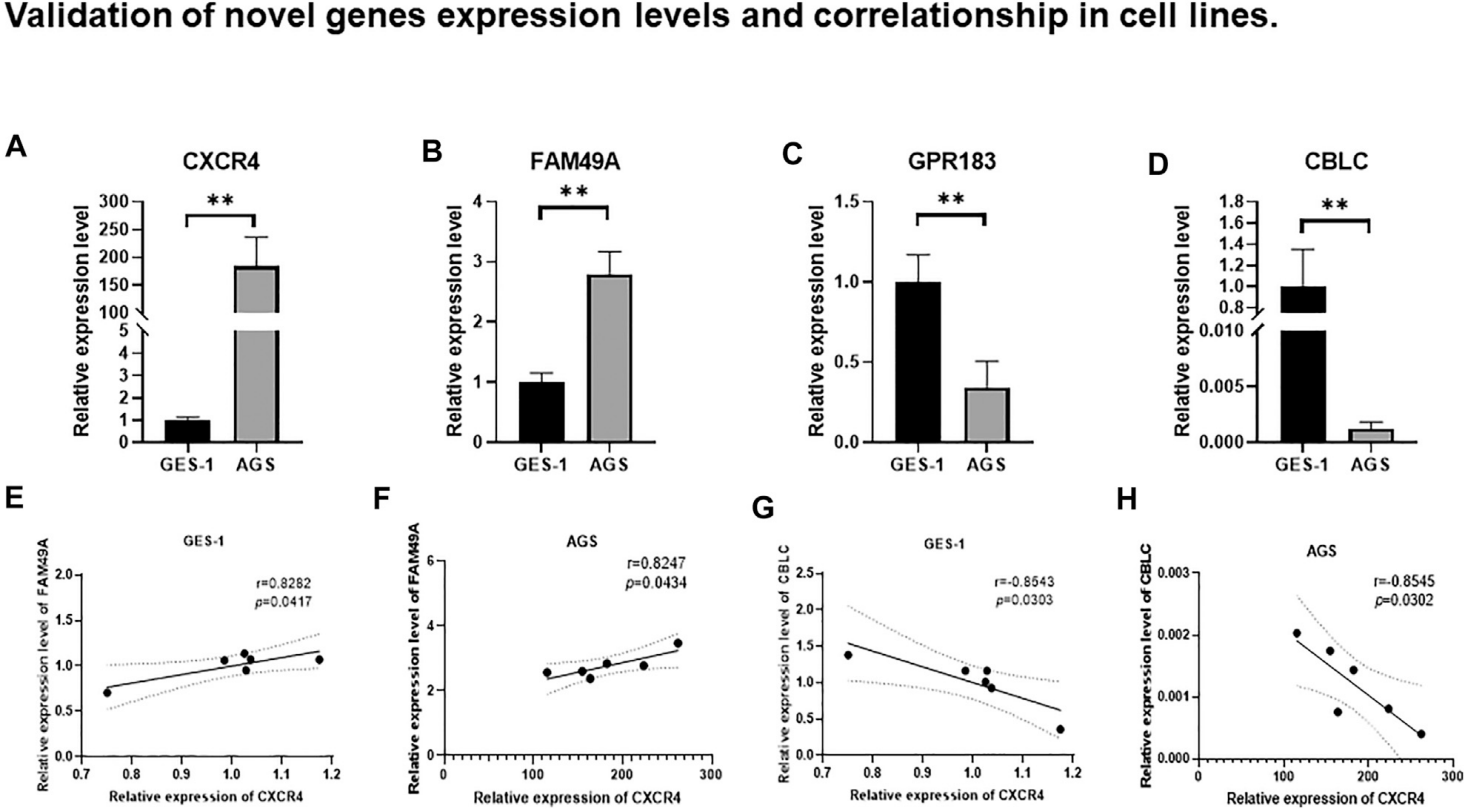
Relative expression levels of **(A)** CXCR4, **(B)** FAM49A, **(C)** GPR183, and **(D)** CBLC between AGS and GES-1. ns: not significant; *p < 0.05; **p < 0.01; ***p < 0.001; ****p < 0.0001. Correlation between CXCR4 and FAM49A in GES-1 **(E)** and AGS **(F)**. Correlation between CXCR4 and CBLC in GES-1 **(G)** and AGS **(H)**.

Among the 19 genes positively correlated with CXCR4, 4 genes (FAM49A, GPR183, CCR7, and GAPT) were identified with the greatest impact on the OS of GC patients according to LASSO Cox regression analysis. FAM49A and GPR183 were selected for further validation. CBLC, the only one gene negatively correlated with CXCR4 in this research, was also sent to validate. The expression levels of CXCR4, FAM49A, GPR183, and CBLC were evaluated in GES-1 and AGS by qRT-PCR. Compared with GES-1, CXCR4 was significantly upregulated in AGS (Figure 8A , *p* < 0.01). For CXCR4 positively correlated genes, FAM49A was significantly upregulated (Figure 8B , *p* < 0.01), while GPR183 was downregulated in AGS (Figure 8C , *p* < 0.01). CBLC, the only one gene negatively correlated with CXCR4, was remarkedly downregulated in AGS (Figure 8D , *p* < 0.01). Consistently, no matter in GES-1 or AGS, FAM49A was positively correlated with CXCR4 (GES-1: r = −0.8545, *p* = 0.0302, Figure 8E; AGS: r = 0.8247, *p* = 0.0434, Figure 8F) and CBLC was remarkedly negatively correlated (GES-1: r = −0.8543, *p* = 0.0303, Figure 8G; AGS: r = −0.8545, *p* = 0.0302, Figure 8H).

## Discussion

Over the past few decades, although the research on gastric tumors itself has received much attention, understanding the mechanism of the process from GPL becoming cancer has made little advances. Besides, biomarkers that can monitor the progression of GPL-to-GC are still much insufficient.

In this study, the expression level and the immunofluorescence positive rate of CXCR4 were observed increasing with GPL progression. Besides, they were significantly different among pathological stages and remarkedly positively correlated with tumorigenesis. Therefore, we identified CXCR4 as a novel biomarker correlated with the malignant transformation of GPL. CXCR4 is a seven-span transmembrane G-protein coupled receptor that is the primary receptor for CXCL12 (Loetscher et al., 1994). CXCR4 plays an important role in tumor biological behaviors, such as growth, metastasis, angiogenesis, and cancer cell–microenvironment interactions (Sleightholm et al., 2017). It is reported that CXCR4 is upregulated in GC and associated with poor prognosis (He et al., 2013; Xu et al., 2020).

We also explored the implication of CXCR4 in GPL malignant transformation. CXCR4 co-expression genes were identified, and they were found to be mainly involved in regulation of actin. The malignant transformation of GPL is accompanied by the enhancement of invasion and metastasis capabilities (Correa and Piazuelo, 2015), which is significantly associated with extracellular matrix (ECM) remodeling (Uloza et al., 2015). The reconstructed ECM forms a loose microenvironment for cancer cells, leading to high proliferation as well as invasion and metastasis of tumor cells (Schedin and Keely, 2011). In ECM remodeling, it is the regulation of actin that serves as the key modulator (Sahai, 2005). Accordingly, from immunofluorescence results, CXCR4 mainly expressed inside the tumor region, which further indicated a crucial role of CXCR4 in the regulation of actin and ECM remodeling in the tumor microenvironment (TME).

CXCR4 co-expression cytokines and immune cells were also identified. CXCR4 co-expression cytokines were mainly enriched in regulation of chemotaxis, cell chemotaxis, mononuclear cell migration, leukocyte chemotaxis, etc. As for co-expression immune cells, we found that the expression level of CXCR4 was positively correlated with the abundance of macrophages but negatively correlated with that of effector memory T cells and NKT cells during GPL malignant transformation. In our research, CXCR4 was observed to be mainly expressed inside the tumor region but rarely in the peri-tumor. It is reported that the SDF-1/CXCR4 axis could attract CXCR4-expressing tumor-associated macrophage (TAM) and myeloid-derived suppressor cell (MDSC) accumulation in TME and suppresses T-cell immune responses (Obermajer et al., 2011; Vatner et al., 2014; Séhédic et al., 2017; Jiang et al., 2019). Therefore, the accumulation of CXCR4-expressing TAMs and MDSCs may be the reason why CXCR4 was mainly observed in tumor areas. Besides, the enhancement of protumor immunity and the impairment of antitumor immunity may result in the malignant transformation of GPL. Last, we constructed a CXCR4-related prognostic model. Our results demonstrated that the risk model was able to predict the prognosis of GC and serve as an independent predictor for OS, which further illustrated that CXCR4 played a vital role in malignant transformation.

Some limitations should be addressed in this study. The mechanisms underpinning CXCR4-medicated malignant transformation of GPL and alteration of tumor immunity should be explored in the future. Second, our model was constructed and validated based on retrospective data. Prospective clinical validation is needed henceforth.

In conclusion, CXCR4 was a novel biomarker correlated with malignant transformation of GPL and played a vital role in the control of tumor immunity. Besides, the prognostic signatures derived from CXCR4-related genes could serve as an independent predictor for OS in GC. CXCR4 is possible to serve as a therapeutic target for malignant transformation of GPL.

## Data Availability

Publicly available data sets were analyzed in this study. These data can be found here: TCGA and the GEO database.
