# Supplementary material for: CXCR4 is a Novel Biomarker Correlated With Malignant Transformation and Immune Infiltrates in Gastric Precancerous Lesions
Source: Front Mol Biosci. 2021 Oct 5;8:697993. doi: 10.3389/fmolb.2021.697993 (PMC8523893; doi:10.3389/fmolb.2021.697993)
Supplement: Supplementary file 1 [file DataSheet1.PDF]

```
library('limma')

workdir="D:\\"
setwd(workdir)
dir.create("result")

files = list.files(workdir, pattern = "GSM")
files
RawData=read.maimages(files=files,source="agilent",green.only = T)
show(RawData)

EListRaw=RawData
dim(EListRaw)
colnames(EListRaw)

BgCorrect=backgroundCorrect(EListRaw,method = "auto",normexp.method = "rma",offset =
50)
BGandNormalized=normalizeBetweenArrays(BgCorrect,method = "quantile")

EList=BGandNormalized

n.Sample=length(files)
SampleNames=apply(as.matrix(files),1,function(x){
  unlist(strsplit(x,"_"))[1]
})
SampleNames

averEList=avereps(EList,ID=EList$genes$ProbeName)
exp=averEList$E
row.names(exp)=averEList$genes$ProbeName
colnames(exp)=SampleNames

write.table(exp, "normalized_expression.xls", sep="\t", row.names=T,quote=F)
```
