# Supplementary figures and images for: CXCR4 is a Novel Biomarker Correlated With Malignant Transformation and Immune Infiltrates in Gastric Precancerous Lesions
Source: Front Mol Biosci. 2021 Oct 5;8:697993. doi: 10.3389/fmolb.2021.697993 (PMC8523893; doi:10.3389/fmolb.2021.697993)

A

GSE55696

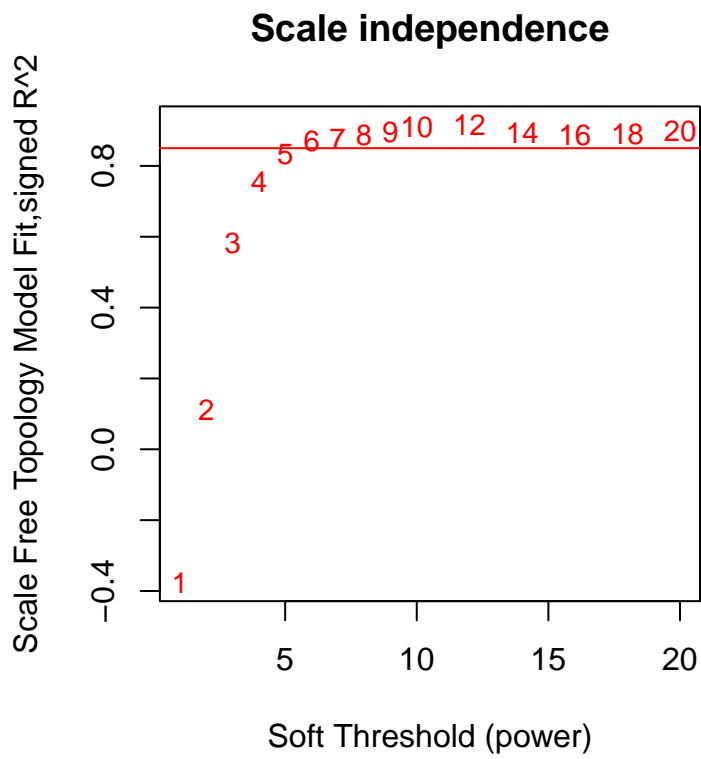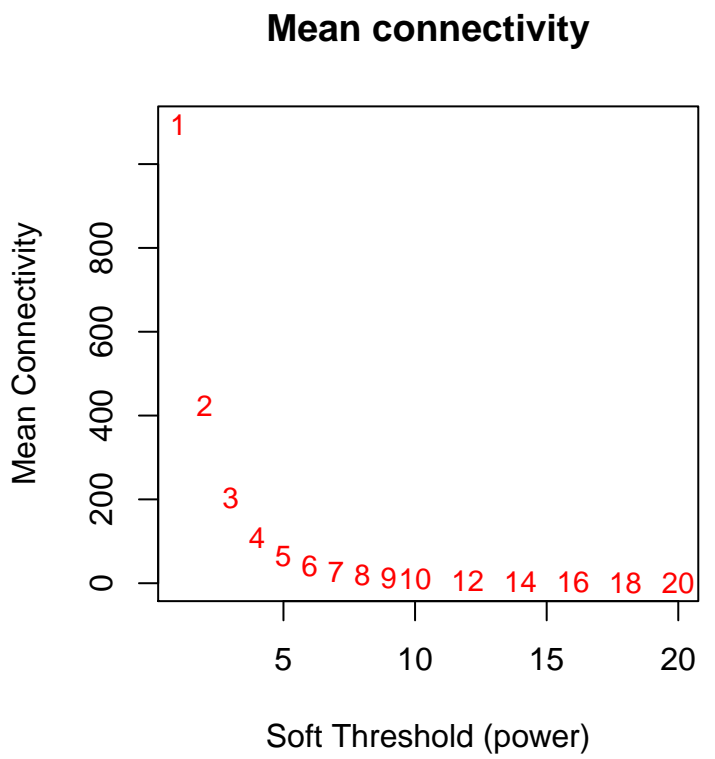

B

GSE130823

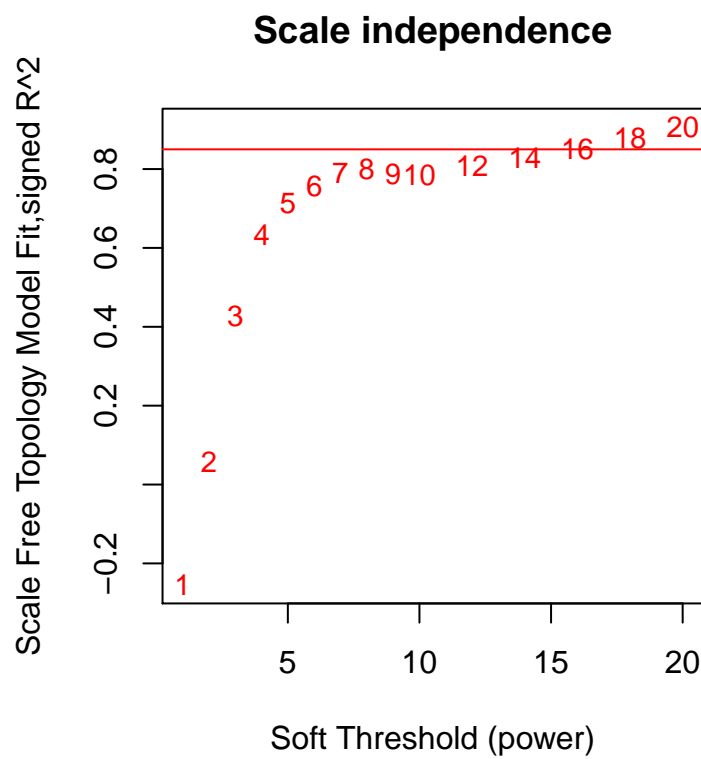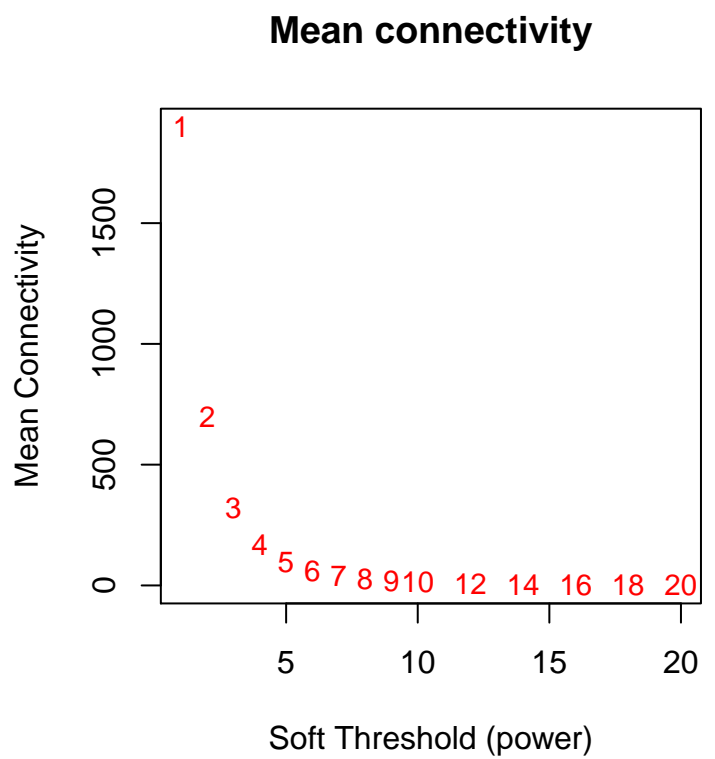

Supplement: Supplementary file 2 [file Image1.pdf]
